# Task-evoked reconfiguration of the fronto-parietal network is associated with cognitive performance in brain tumor patients

**DOI:** 10.1007/s11682-019-00189-2

**Published:** 2019-08-27

**Authors:** Wouter De Baene, Martijn J. Jansma, Irena T. Schouwenaars, Geert-Jan M. Rutten, Margriet M. Sitskoorn

**Affiliations:** 1 Department of Cognitive Neuropsychology, Tilburg University, Warandelaan 2, PO Box 90153, 5000 LE Tilburg, The Netherlands; 2 Department of Neurosurgery, Elisabeth-TweeSteden Hospital, Tilburg, Netherlands

**Keywords:** Task-evoked network reconfiguration, Fronto-parietal network, Brain tumor patients, Working memory, Cognitive flexibility

## Abstract

In healthy participants, the strength of task-evoked network reconfigurations is associated with cognitive performance across several cognitive domains. It is, however, unclear whether the capacity for network reconfiguration also plays a role in cognitive deficits in brain tumor patients. In the current study, we examined whether the level of reconfiguration of the fronto-parietal (‘FPN’) and default mode network (‘DMN’) during task execution is correlated with cognitive performance in patients with different types of brain tumors. For this purpose, we combined data from a resting state and task-fMRI paradigm in patients with a glioma or meningioma. Cognitive performance was measured using the in-scanner working memory task, as well as an out-of-scanner cognitive flexibility task. Task-evoked changes in functional connectivity strength (defined as the mean of the absolute values of all connections) and in functional connectivity patterns within and between the FPN and DMN did not differ significantly across meningioma and fast (HGG) and slowly growing glioma (LGG) patients. Across these brain tumor patients, a significant and positive correlation was found between the level of task-evoked reconfiguration of the FPN and cognitive performance. This suggests that the capacity for FPN reconfiguration also plays a role in cognitive deficits in brain tumor patients, as was previously found for normal cognitive performance in healthy controls.

## Introduction

In Europe, between 8.5 and 14 per 100,000 persons per year are diagnosed with a primary brain tumor (Gigineishvili et al. 2014). In adults, the most common types of primary brain tumours are gliomas, developing from glial cells, and meningiomas, developing in the meninges. Gliomas are infiltrating tumors that lack a clear boundary between normally functioning brain tissue and pathological tumor tissue. They are classified based on their malignancy: Low-grade gliomas (LGG) tend to grow more slowly and less aggressively with lower degrees of cell infiltration and proliferation compared to high-grade gliomas (HGG). Meningiomas, by contrast, only infrequently infiltrate the surrounding brain tissue and most of them have a benign clinical course (Ostrom et al. 2016).

The majority (up to 90%) of patients with a primary brain tumor show cognitive deficits (Gehring et al. 2008) across multiple domains (e.g. memory, attention, information processing, executive functioning; Gehring et al. 2012). These cognitive deficits can be very disruptive for a person’s daily functioning, experienced quality of life and treatment compliance (Talacchi et al. 2011; Taphoorn et al. 2010). Cognitive deficits are present across different tumor types (Klein et al. 2003; Meskal et al. 2016, 2015; Miotto et al. 2011; Tucha et al. 2001), despite the fact that meningioma, low-grade glioma and high-grade glioma patients have distinct prognoses.

Complex cognitive functions that involve multiple forms of information processing depend on interactions across a collection of brain areas. These brain areas are organized into large-scale networks (Bressler and Menon 2010; Gratton et al. 2016; Power et al. 2011; Smith et al. 2009). Based on functional connectivity measurements, it appears that the spatial architecture of these networks is fairly stable across diverse cognitive domains as well as across task execution and rest (e.g. Betti et al. 2013; Cole et al. 2014; Gratton et al. 2016; Krienen et al. 2014). The stability of these intrinsic networks over rest and task execution points to an intrinsic topology, possibly resulting from the structural connectivity between, and the longlasting coactivation of regions across the lifespan (Dosenbach et al. 2007).

Notwithstanding their stable spatial architecture across rest and task, these networks can also show modest but reliable task-specific changes in functional connectivity. These task-evoked changes seem to reflect specific task demands (Bullmore and Sporns 2012; Cole et al. 2013; Krienen et al. 2014), suggesting that the brain adjusts its connectivity to facilitate task execution (Cole et al. 2014). This has been shown across a wide range of domains, amongst others, for reasoning (Cocchi et al. 2014; Hearne et al. 2015), working memory (Braun et al. 2015; Vatansever et al. 2017, 2015), and cognitive control (Cocchi et al. 2013; Dwyer et al. 2014).

Importantly, in healthy subjects, the level of task-evoked network reconfigurations has been associated with the variance in cognitive performance in several cognitive domains, such as learning (Bassett et al. 2011), working memory (Braun et al. 2015; Vatansever et al. 2017; Vatansever et al. 2015), attention (Shine et al. 2016), cognitive control (Dwyer et al. 2014) and general intelligence (Schultz and Cole 2016). The direction of this relation, however, is not very clear yet. Some studies have found that individuals with greater network reconfiguration when performing the task show enhanced cognitive performance (e.g. Braun et al. 2015; Tommasin et al. 2018). This is in line with the idea that larger changes in the functional connectivity pattern are associated with larger, more optimal updates from rest, leading to improved behavioral performance. Alternatively, smaller changes in functional connectivity patterns between rest and task have also been associated with better behavioral performance (e.g. Schultz and Cole 2016; Zuo et al. 2018). This has been explained by the fact that high-performing individuals have a more optimal network organization at rest, requiring less effort to update the functional connectivity pattern to a state that is optimal to perform the task.

Currently, even less is known about network reconfiguration during task performance in brain tumor patients. One may argue that impaired network reconfiguration is related to the broad range of cognitive deficits that are frequently seen in these patients. Identification of these underlying network dynamics of cognitive deficits could therefore be an important first step towards development of clinical biomarkers for prognosis and treatment-response of cognitive functions (Derks et al. 2014). The goal of this study was therefore to explore whether task-evoked network reconfigurations are also associated with cognitive functioning in brain tumor patients. Although cognitive dysfunctions manifest themselves across multiple domains in brain tumor patients, executive functioning is one of the most frequently affected domains (Habets et al. 2014; Noll et al. 2015). As measures of cognitive performance, we therefore looked at tasks that are considered to strongly engage executive functions, more specifically an N-back task that assesses working memory function and an out-of-scanner shifting attention task that assesses cognitive flexibility.

Both working memory and cognitive flexibility involve multiple brain networks, most importantly the fronto-parietal (‘FPN’) and the default mode network (‘DMN’) (e.g. Braver et al. 2003; De Baene et al. 2012; Dosenbach et al. 2007; Douw et al. 2016; Gordon et al. 2012; Provost and Monchi 2015; Repovs and Barch 2012; Spreng et al. 2014; Yin et al. 2018). Several studies have reported an association between the level of task-evoked reconfiguration of both the FPN and the DMN and cognitive performance on a working memory task (Schultz and Cole 2016; Tommasin et al. 2018; Zuo et al. 2018). Furthermore, the cooperation between DMN and FPN seems to be critical to perform challenging cognitive tasks (Cocchi et al. 2013). For executive functioning, increased connectivity between DMN and FPN has been associated with better performance (e.g. Bluhm et al. 2011; Dwyer et al. 2014; Fornito et al. 2012).

To investigate the link in brain tumor patients between task-induced reconfiguration of the FPN and DMN and working memory performance, we used an N-back working memory paradigm in the scanner to evoke reconfigurations in brain activity. Furthermore, we examined whether the link between task-induced reconfiguration and cognitive performance also generalizes to another executive function, namely cognitive flexibility that was assessed outside the scanner and consequently, did not have a direct causal relationship with the reconfigurations in brain activity measured in the scanner.

Given that task-evoked functional connectivity changes of the FPN and DMN are associated with cognitive performance in healthy subjects, we also expected a similar association between reconfiguration of these networks and executive function abilities in brain tumor patients.

## Methods and procedure

### Study population

All newly diagnosed meningioma and glioma patients undergoing resective tumor surgery at the Elisabeth-Tweesteden Hospital (Tilburg, the Netherlands) between July 2016 and August 2018 were eligible for participation. Inclusion criteria were, next to histologically confirmed unilateral glioma WHO grade II-IV or meningioma, the availability of (1) resting state fMRI, (2) task fMRI, (3) structural 3D MRI necessary for co-registration, and (4) neuropsychological test results. Exclusion criteria were (1) history of intracranial neurosurgery, (2) history of cranial radiotherapy or chemotherapy, and (3) history of neurological or psychiatric disorders.

This study was approved by the Medical Ethics Committee Brabant, The Netherlands [protocol number: NL51147.028.14]. All procedures were carried out with written informed consent of all subjects and in accordance with the principles of the Declaration of Helsinki.

### Experimental procedure

One to five days before the tumor resection, patients were neuropsychologically assessed and scanned. In this scan session, we collected anatomical, resting state and task data.

### Neuropsychological assessment

As a measure of cognitive flexibility, we used the results on the shifting attention task that is part of the Central Nervous System Vital Signs (CNS VS; Gualtieri and Johnson 2006). The CNS VS is a brief computerized battery composed of seven neuropsychological tests (for more details on the different tests, see Rijnen et al. 2017). The CNS VS takes approximately 30 to 40 min to complete and generates 11 cognitive domain scores. The results on the shifting attention task (#correct responses - #incorrect responses) are summarized in the scores on the “executive functioning” domain. Higher executive functioning scores therefore reflect better performance.

### In-scanner task design

The N-back task was part of a larger fMRI experiment with multiple conditions with varying working memory load. Each of the conditions consisted of 2 blocks. The task was presented in blocks of 30 s, with two or three consecutive conditions, interleaved with rest blocks of 15 s. Instructions for each condition were presented for four seconds prior to the relevant task block.

In the N-back task, patients payed attention to a fast sequence of consonants: Stimuli were presented for 400 ms with an inter-stimulus interval of 1 s at the center of the screen. Patients needed to respond if a stimulus was equal to a stimulus presented 2 trials before (i.e. a 2-back task) by pushing a button on a button box with their right hand.

### (f)MRI acquisition

Subjects were positioned head first and supine in the magnetic bore. Images were collected with a 3 Tesla Philips Achieva scanner (Philips Medical Systems, Best, The Netherlands) using a standard 32-channel radiofrequency head coil. Participants were instructed not to move their heads in order to avoid motion artefacts. First, high-resolution whole-brain anatomical images were acquired using a T1-weighted sequence for anatomical registration purposes (TR/TE: 8.4/3.8 ms, FOV: 254x254x158 mm, flip angle: 8°, sagittal slice orientation, voxel size 1 mm isotropic). Task and resting state fMRI volumes were obtained using an EPI pulse sequence (TR/TE: 2000/28 ms, transverse slice orientation, FOV: 240x240x111 mm, voxel size: 3x3x3). A fixed number of 219 task functional volumes and 225 resting state volumes were collected per patient. During the resting state scan, all subjects were instructed to close their eyes and relax, but not to sleep, in the scanner while thinking of nothing in particular.

### MRI data pre-processing

Imaging data were analysed using SPM12 (Wellcome Trust Center for Neuroimaging, London, UK) and the CONN-toolbox (Whitfield-Gabrieli and Nieto-Castanon 2012). Both resting state and task data were preprocessed with the same pipeline. Since tumor tissue may theoretically alter the BOLD response locally and confound our connectivity analyses, we excluded voxels covered by the tumor mask on a subject level.

Preprocessing included realignment, slice time correction, functional outlier detection (based on ART-based scrubbing with a global-signal scan-to-scan Z-value threshold of 3 and a composite motion-value threshold of 0.5 mm), segmentation of the structural image, spatial normalization of the structural and functional images to the template MNI brain, resampling to 2 × 2 × 2 mm cubic voxels and smoothing using a 4 mm full width at half maximum (FWHM) Gaussian Kernel.

Possible sources of spurious variance were regressed out from the data, including (a) undesired linear trends, (b) the realignment and scrubbing parameters, (c) the white matter signal, and (d) the ventricular system signal. Global signal regression was not performed due to the ongoing controversy associated with this step (Caballero-Gaudes and Reynolds 2017; Saad et al. 2012). To allow comparison of resting state and task data, filtering (0.01–0.15 Hz) at a low frequency component of the BOLD signal known to be sensitive to both resting state and task-based functional connectivity (Bassett et al. 2015; Sun et al. 2004) was also applied.

Given that task-evoked activations substantially inflate task-state functional connectivity estimates (Al-Aidroos et al. 2012; Cole et al. 2019, 2013; Fair et al. 2007), we removed these task-evoked activations before calculating the functional connectivity of the task fMRI time series (see below) using the finite impulse response (FIR) task regression approach put forward by Cole et al. (2019). This approach allows to empirically determine the correct HRF shape for task regression by fitting the cross-trial mean response for each time point in a specific time window that is time-locked to the trial onset for a given task condition (Fair et al. 2007). Each task condition (separately for targets and non-targets) was therefore fit with a series of 10 regressors, one per time window (resulting in a time window of 20 s), to account for the likely duration of the HRF. The residual time series from this regression were used for task functional connectivity estimation.

### Functional connectivity

**Fig. 1:**
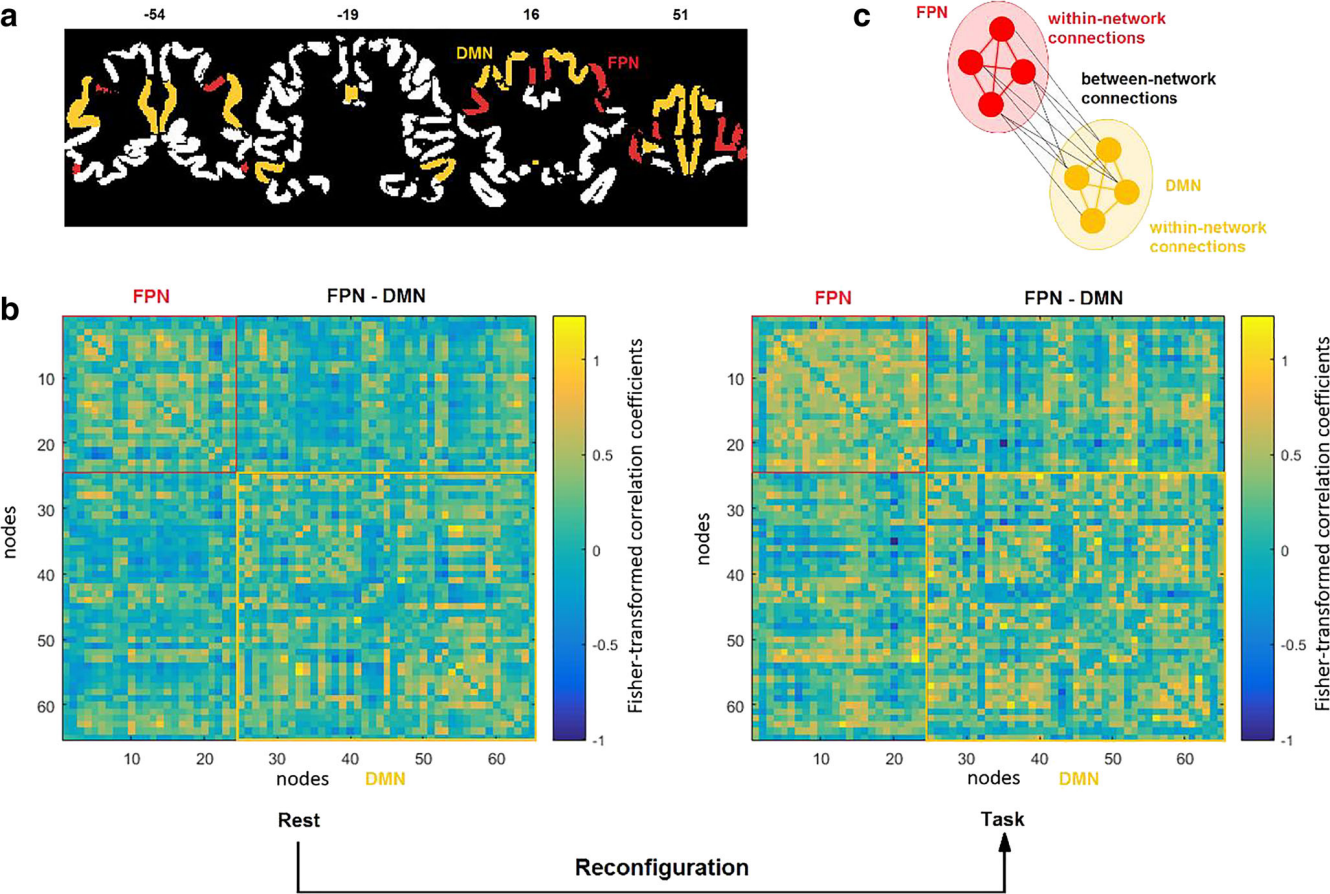
**a** FPN (in red) and DMN (in yellow) regions defined according to the Gordon parcellation. **b** Correlation matrix for one subject in rest (left) and during the N-back task (right). Nodes 1 to 24 belong to the FPN network. Nodes 25 to 65 belong to the DMN network. **c** Definition of within- and between-network connections. Connections between nodes of the FPN (in red) and connections between nodes of the DMN (in yellow) are within-network connections. Connections between nodes of the FPN and nodes of the DMN (in black) are between-network connections

To assess the functional connectivity in each patient, preprocessed rs-fMRI and task data were first parcellated into 333 regions of interest (ROIs) according to the Gordon parcellation scheme (Gordon et al. 2016). These 333 parcels divide into 12 different functional brain networks, including the cingulo-opercular (CON), salience (SN), fronto-parietal (FPN), dorsal attention (DAN), ventral attention (VAN) and default mode (DMN) networks. The representative time series for each ROI were obtained by averaging the BOLD time series over the tumor-free extent of the parcel. For each patient and for both rest and task data, a weighted adjacency matrix was created by computing the correlation coefficient between every pair of nodes which were then Fisher transformed. The fully-weighted functional connectivity values were used rather than the binarized ones to conserve all connectivity information (Bassett et al. 2011; Vatansever et al. 2015; Zuo et al. 2018). From this correlation matrix (see Fig. 1a, b) we extracted nodes from our a priori networks to create network-specific graphs for the FPN (24 × 24) and the DMN (41 × 41). Additionally, we created an FPN-DMN graph (24 × 41) containing all connections between the 24 FPN regions and the 41 DMN regions. Consequently, we ended up with six (3 networks × 2 task states) graphs for each subject. Regions of the FPN or DMN that were fully covered by tumor mask on a subject level were excluded for that patient from our analyses. The resulting number of missing regions within the FPN or within the DMN due to tumor overlap was used as a covariate in our analyses.

To quantify the communication abilities within and between networks, we computed the connection strength (Zuo et al. 2018) for each of these six graphs (see Fig. 1c). This connection strength is defined as the mean of the absolute values of all connections in a graph. As a measure of change from rest to task, we computed the ratio between the connection strength for the task graphs and the connection strength for the rest graphs.

Task-evoked changes in functional connectivity strength are not necessarily identical across all connections within a network or across all between-network connections (e.g. Vatansever et al. 2017). The connection strength measure used here is not able to dissociate these connection-specific changes. Therefore, we also examined task-evoked changes in the functional connectivity patterns within the FPN and DMN as well as between these two networks. To this end, as a first measure of similarity of functional connectivity patterns between task and rest, we computed the Pearson correlation between the network graphs in resting state and in task state for each patient and for each of the networks (Schultz and Cole 2016). For the FPN and DMN, we excluded redundant edges by considering only the upper triangle of the network graph. For the FPN-DMN graph, all edges were considered. The resulting correlation coefficients were Fisher transformed for each network. Lower values indicate stronger task-evoked reconfiguration based on the inference that lower similarity in functional connectivity patterns between rest and task are thought to indicate that a larger distance in state-space is required to travel from one condition to another condition.

As a second measure of similarity of functional connectivity patterns between task and rest, we computed the slope (β_1_) of the general linear model between task connectivity and rest connectivity (i.e. task connectivity = β_1_ * rest connectivity + β_0_) for the FPN, DMN and FPN-DMN graph (Tommasin et al. 2018). Again, for the FPN and DMN, we excluded redundant edges by considering only the upper triangle of the network graph. For the FPN-DMN graph, all edges were considered. Lower values for the slope indicate a reduced linear dependence of connectivity in the task state versus connectivity in the resting state, thus a higher task-evoked reconfiguration.

### Statistical analyses

Pearson’s Chi square tests were performed to test for differences between groups (between meningioma, LGG and HGG patients) in sex, education, tumor hemisphere and frontal versus non-frontal tumor involvement. Kruskal-Wallis tests were performed to explore group differences in age, tumor volume, missing regions within the FPN (due to tumor overlap) and missing regions within the DMN (due to tumor overlap). Since even subtle head movement during a scan can spuriously affect measures of functional connectivity (Power et al. 2012; Van Dijk et al. 2012), we also checked the group differences in head motion during resting state and during task state according to the composite motion score and according to the number of time points scrubbed. When a Kruskal-Wallis test showed significant results (*p* < 0.05), post-hoc analyses were performed by means of Mann-Whitney U tests.

We tested patient group differences in cognitive performance (accuracy on the N-back task and raw scores on the cognitive flexibility task) using linear mixed-models (using the *fitlme* function in MATLAB R2016a). To estimate the model parameters, the maximum likelihood estimation method was used. An unstructured covariance matrix was used in which all elements of the variance-covariance matrix are estimated (Cholesky parametrization). In every model, subject-ID was modelled as a random effect and the variables patient group (dummy coded; meningioma as reference category), age (in years), sex, education (dummy coded; middle education as reference category), tumor hemisphere, frontal versus non-frontal tumor involvement and tumor volume (in cm^3^) were included as fixed effects in the model.

To test for patient group differences in similarity of functional connectivity patterns between resting state and task state, these linear mixed-models (one for each network) were extended with the addition of the number of missing regions within the respective network as a fixed factor. Note that for the FPN-DMN network, the number of missing regions within both the FPN and the DMN were added as fixed factors.

To test for differences in connection strength between resting state and task state, the linear mixed-models (one for each network) were further extended by adding state (resting state vs task state) as a fixed factor. In a second step, these models were even further extended to account for interactions between state and patient group to test for group differences in task-evoked changes in connection strength.

To evaluate whether the similarity of functional connectivity patterns between resting state and task state accounts for a substantial proportion of individual variability in cognitive performance, the linear-mixed models for working memory and for cognitive flexibility performance were further expanded. For both performance measures and separately for the different networks, the Fisher-transformed correlation coefficients between the resting and task state graphs of that particular network or the slope of the linear model between task connectivity and rest connectivity were added to the model as predictor variables. Similarly, to evaluate whether the task-evoked change in connection strength accounts for a substantial proportion of individual variability in cognitive performance, the ratio between the connection strength for the task graph and the connection strength for the rest graph was added to the linear-mixed models as predictor variable. In all these models, the ratio between the composite motion score in task state versus resting state was included as fixed factor.

For each of the linear-mixed models described above, we next performed a backward elimination analysis to develop the most parsimonious model (Heinze et al. 2018). In this analysis, the weakest (sociodemographic, clinical and control) variables are sequentially eliminated until only those making a statistically significant contribution to the model (*p* < .05) remain. Note that similar results were found when all variables were included in the model.

A significance threshold of α = 0.05 was used. The false discovery rate (FDR) correction was applied for multiple comparisons. FDR-adjusted *p*-values are reported where necessary.

## Results

### Subject information and behavioral performance

**Fig. 2:**
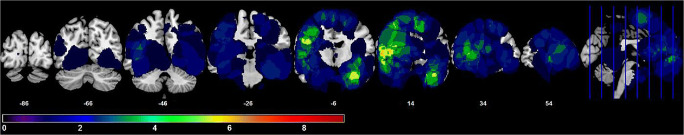
Frequency distribution of tumor (all 48 patients). The color scale shows minimal overlap (dark blue) to maximal overlap (red). MNI y coordinates of the coronal sections are given

The initial sample contained 53 patients. Based on the functional outlier detection, one patient was excluded from further analyses because too little time points remained in the task state (43% of all task state time points were scrubbed for this patient). One patient did not have valid scores on the cognitive flexibility task. One patient scored more than 2.5 standard deviations below the mean on the N-back task, 2 patients scored more than 2.5 standard deviations below the mean on the cognitive flexibility task. All these patients were removed from all further analyses. Consequently, 48 patients were included in the final data analyses. The distribution of the tumors across these 48 patients is shown in Fig. 2.

Detailed sociodemographic and clinical information about the patients is listed in Table 1.

**Table 1:** Sociodemographical and clinical characteristics

Variable	All patients (*n* = 48)	Meningioma patients (*n* = 22)	LGG patients (*n* = 13)	HGG patients (n = 13)
Female (n)	26 (54.17%)	16 (72.73%)	3 (23.08%)	7 (53.85%)
Age in years (mean; range)	48.64 (18–73)	53.00 (32–73)	41.23 (21–67)	48.69 (18–68)
Tumor volume in cm^3^ (mean; range)	41.98 (2.56–148.42)	32.87 (2.56–92.05)	40.08 (4.84–97.13)	59.29 (13.11–148.42)
Education (n)				
Low (Verhage 1–4)	9 (18.75%)	4 (18.18%)	3 (23.08%)	2 (15.38%)
Middle (Verhage 5)	16 (33.33%)	9 (40.91%)	3 (23.08%)	4 (30.77%)
High (Verhage 6–7)	23 (47.92%)	9 (40.91%)	7 (53.85%)	7 (53.85%)
Left tumor hemisphere (n)	27 (56.25%)	12 (54.55%)	7 (53.85%)	8 (61.54%)
Frontal tumor involvement (n)	31 (64.58%)	17 (77.27%)	10 (76.92%)	4 (30.77%)
Missing regions within FPN in % (mean; std)	1.48 (4.08)	1.70 (4.58)	0.96 (2.50)	1.60 (4.67)
Missing regions within DMN in % (mean; std)	0.97 (2.83)	1.00 (2.88)	0.38 (1.35)	1.50 (3.80)
Head motion resting state (mean; std)	0.20 (0.08)	0.20 (0.08)	0.23 (0.11)	0.18 (0.06)
Head motion task state (mean; std)	0.13 (0.04)	0.12 (0.05)	0.14 (0.04)	0.12 (0.03)
Scrubbed time points resting state in % (mean; std)	6.78 (8.72)	6.08 (7.47)	9.95 (12.56)	4.82 (5.11)
Scrubbed time points task state in % (mean; std)	7.64 (8.70)	8.33 (7.82)	8.21 (11.19)	5.90 (7.72)

The group of 48 patients consisted of 22 patients with a meningioma, 13 patients with a LGG (including 7 astrocytoma and 6 oligodendroglioma) and 13 patients with a HGG (including 2 IDH-wildtype LGG, 1 secondary glioblastoma and 10 primary glioblastoma).

To classify the level of education, the Dutch Verhage scale was used (Verhage 1964). Its seven categories were merged into three ordinal categories: low (Verhage 1–4), middle (Verhage 5), and high educational level (Verhage 6 and 7)(Cf. Rijnen et al. 2017).

There were no significant group differences in age (Kruskal-Wallis Chi square = 4.8; *p* = .09), tumor volume (Kruskal-Wallis Chi square = 1.28; *p* = .53), educational level (Chi square = 1.44; *p* = .84), tumor hemisphere (Chi square = 0.20; *p* = .90), missing regions within the FPN (Kruskal-Wallis Chi square = 0.34; *p* = .85), missing regions within the DMN (Kruskal-Wallis Chi square = 0.5; *p* = .78), head motion during resting state (Kruskal-Wallis Chi square = 1.57; *p* = .46), head motion during task state (Kruskal-Wallis Chi square = 2.63; *p* = .27), number of time points scrubbed from resting state data (Kruskal-Wallis Chi square = 2.6; *p* = .27) or number of time points scrubbed from task state data (Kruskal-Wallis Chi square = 0.9; *p* = .64). A significant difference in sex (Chi square = 8.11; *p* < .05) and in frontal versus non-frontal tumor involvement (Chi square = 8.91; *p* < .05) was found between groups.

**Fig. 3:**
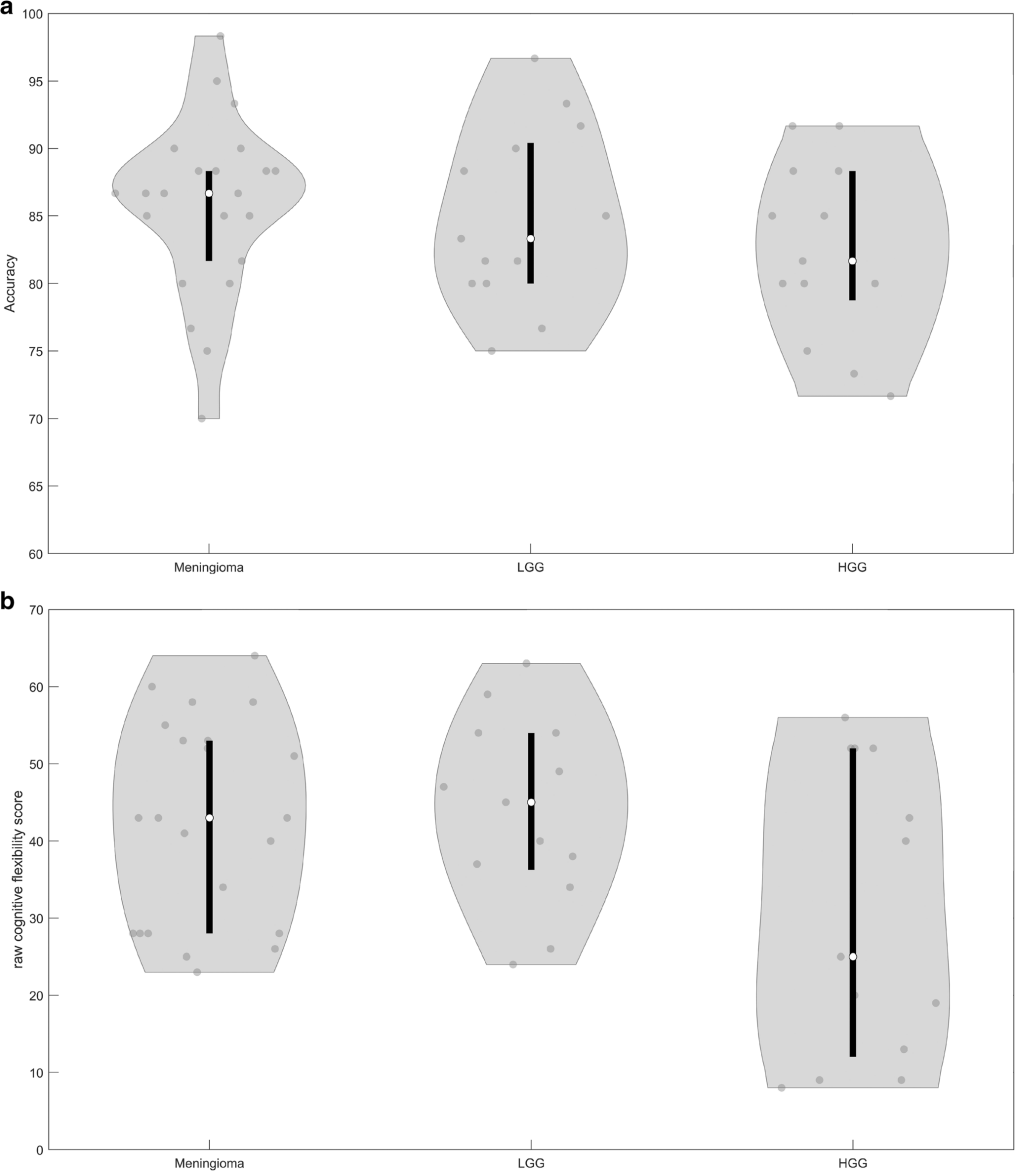
Distribution of the results on (**a**) the N-back task and (**b**) the cognitive flexibility task for the meningioma (left column), LGG (middle column) and HGG (right column) patients. The contour of the violin plot represents the estimate of the density of patients with particular performance scores. The filled circles represent the individual data points. White circles and black line segments denote, respectively, the median and 1st and 3rd quartiles

Taking sex into account (the only variable that reached statistical significance after backward elimination; F(1,44) = 4.92, *p* < .05), the accuracy on the N-back task was not significantly different between groups (Meningioma: mean = 85.68%; LGG: mean = 84.87%; HGG: mean = 82.44%; F(2,44) = 1.26, *p* = .29). The performance on the shifting attention task, however, did differ between groups (Meningioma: mean score = 42.45; LGG: mean score = 43.85; HGG: mean score = 30.62; F(2,44) = 7.13, *p* < .05), taking frontal versus non-frontal tumor involvement into account (F(1,44) = 7.40, *p* < .05). Post hoc analyses, however, showed no significant pairwise differences (all *p*’s > .11 after FDR correction for multiple testing), see Fig. 3.

Performance on the N-back task correlated significantly with the cognitive flexibility performance (Spearman rank correlation = .33, *p* < .05). Furthermore, behavioral performance was not correlated with the composite motion score. Both performance on the N-back task and performance on the cognitive flexibility task were not correlated with task state motion (Spearman rank correlation = −.20, *p* = .18 for N-back task; Spearman rank correlation = −.03, *p* = .84 for cognitive flexibility task) or with resting state motion (Spearman rank correlation = −.18, *p* = .22 for N-back task; Spearman rank correlation = −.07, *p* = .64 for cognitive flexibility task).

### Functional connectivity differences between rest and task

**Fig. 4:**
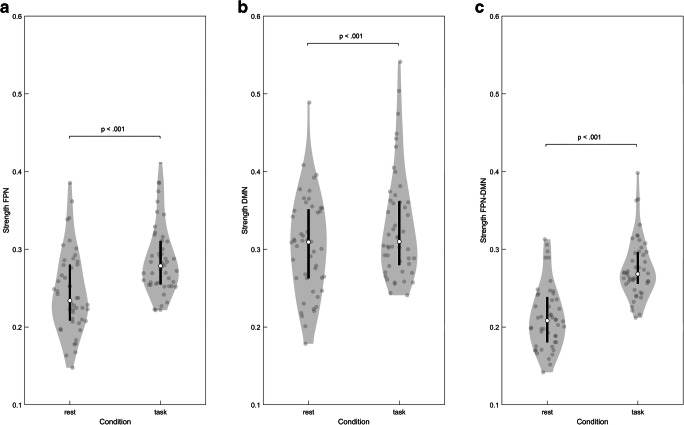
Distribution of the connection strength of (**a**) the FPN, (**b**) the DMN and (**c**) the FPN-DMN across the three tumor groups for the resting state (left part of the graphs) and for the task state (right part of the graphs). The contour of the violin plot represents the estimate of the density of patients with particular connection strength. The filled circles represent the individual data points. White circles and black line segments denote, respectively, the median and 1st and 3rd quartiles

Linear mixed effects models were used to test for connection strength differences between resting and task state for the different networks, taking age, educational level, sex, tumor volume, tumor hemisphere, frontal versus non-frontal tumor involvement and missing regions within the respective networks (FPN, DMN or both) into account. After backward elimination, only tumor volume remained as fixed effect in the FPN model (F(1,93) = 5.30, *p* < .05). For the DMN and FPN-DMN, no sociodemographic, clinical and control variables remained in the model after backward elimination. Connection strength was larger during task relative to rest (after FDR correction for multiple testing) for the FPN (F(1,93) = 673.26; p < .001), DMN (F(1,94) = 365.99, *p* < .001) and FPN-DMN (F(1,94) = 808.81, p < .001), see Fig. 4.

### Effect of tumor type on task-evoked network reconfiguration

To examine differences between meningioma, LGG and HGG patients on the changes in connection strength between rest and task, the interaction between patient group and state was added to the linear mixed effects models, taking also age, educational level, sex, tumor volume, tumor hemisphere, frontal versus non-frontal tumor involvement and missing regions within the respective networks (FPN, DMN or both) into account. After backward elimination, the final models contained the main effects of state and patient group as well as their interaction. Furthermore, tumor volume remained as fixed effect in the FPN model (F(1,89) = 5.91, *p* < .05). Tumor type did not modulate the task-evoked changes in connection strength (after FDR correction) in the FPN (F(2,89) = 1.12, *p* = .42), DMN (F(2,90) = 1.66, *p* = .42) or FPN-DMN (F(2,90) = 0.87, *p =* .42).

**Fig. 5:**
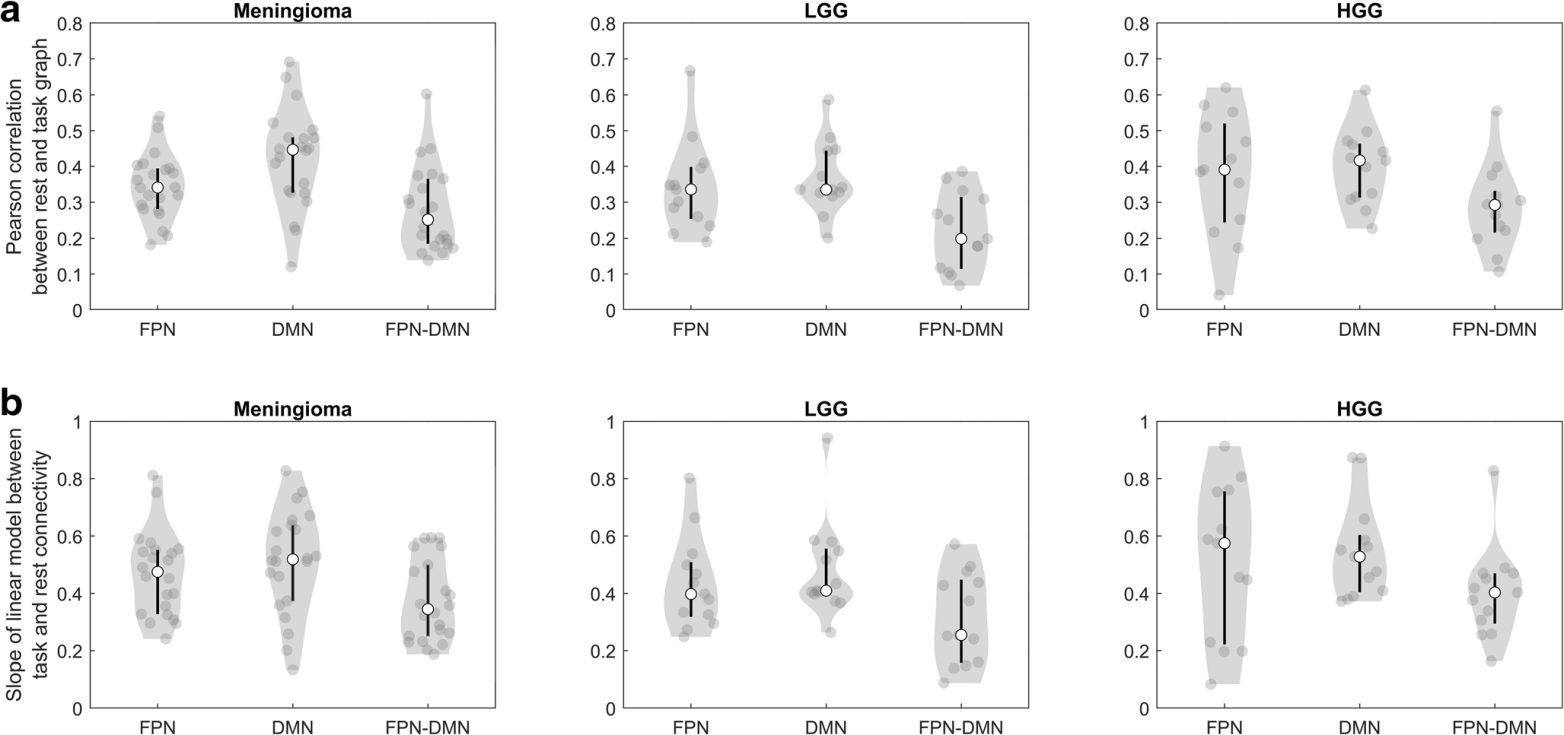
Distribution of the similarity of functional connectivity patterns between rest and task of the different networks for the meningioma (left graph), LGG (middle graph) and HGG (right graph) patients. Top row: Pearson correlation between rest and task graph. Bottom row: slope of linear model between task and rest connectivity. The contour of the violin plot represents the estimate of the density of patients with particular connection strength. The filled circles represent the individual data points. White circles and black line segments denote, respectively, the median and 1st and 3rd quartiles

To examine differences between the different tumor types in the two measures of similarity of functional connectivity patterns between rest and task, a linear mixed effects model was defined per measure for each network, taking age, educational level, sex, tumor volume, tumor hemisphere, frontal versus non-frontal tumor involvement and missing regions within the respective networks (FPN, DMN or both) into account. For the Pearson correlation between the network graphs, only tumor side remained as fixed effect in the FPN-DMN model (F(1,44) = 10.19, *p* < .05). None of the sociodemographic, clinical and control variables remained in the FPN and DMN model after backward elimination. Tumor type did not affect (after FDR correction) the level of task-evoked network reconfiguration of the FPN (F(2,45) = .45, *p* = .64), the DMN (F(2,45) = .85, *p* = .64) or the FPN-DMN (F(2,44) = 2.01, *p* = .44)(Cf. Fig. 5a). For the slope of the general linear model between task connectivity and rest connectivity, the following variables remained as fixed effect in the final model after backward elimination: tumor volume in the FPN model (F(1,44) = 8.57, *p* < .05), missing regions within the DMN in the DMN model (F(1,44) = 4.67, *p* < .05) and tumor side in the FPN-DMN model (F(1,44) = 6.45, *p* < .05). Tumor type did not affect (after FDR correction) the level of task-evoked network reconfiguration of the FPN (F(2,44) = .34, *p* = .75), the DMN (F(2,44) = .29, *p* = .75) or the FPN-DMN (F(2,44) = 1.65, *p* = .61) (Cf. Fig. 5b).

**Fig. 6:**
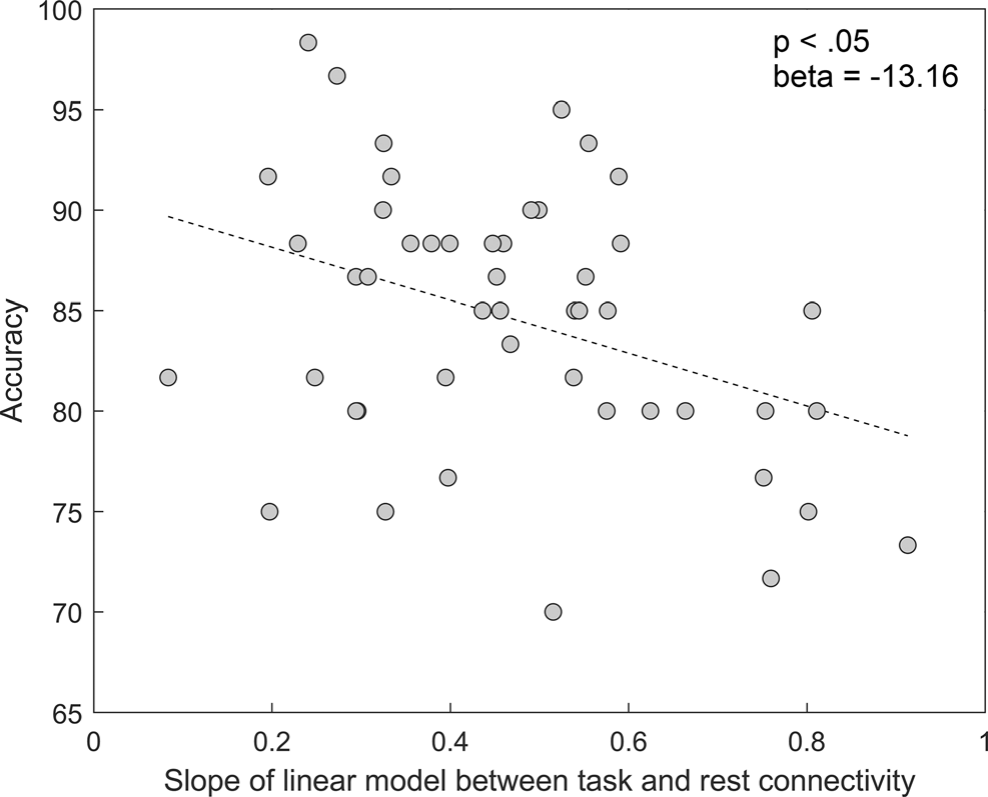
Association between Working Memory performance and the slope linking connectivity during task and at rest for the FPN network (after adjusting for the effect of sex)

**Fig. 7:**
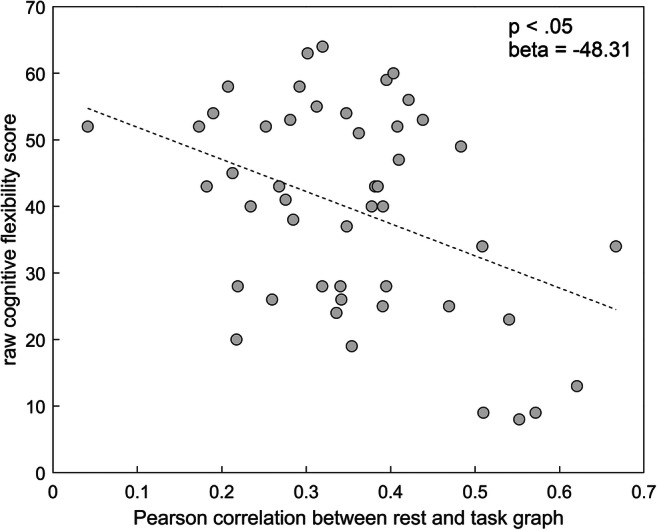
Association between Cognitive Flexibility performance and the Pearson correlation between rest and task graph for the FPN network (after adjustment for the effect of frontal tumor involvement, tumor group and tumor hemisphere)

### Behavioral relevance of functional connectivity differences between rest and task

Separately for both performance measures (working memory and cognitive flexibility performance) and for every network (FPN, DMN and FPN-DMN), a linear mixed model with the similarity of functional connectivity patterns between resting state and task state as a predictor variable (on top of the variables age, educational level, sex, tumor type, tumor volume, tumor hemisphere, frontal versus non-frontal tumor involvement, missing regions within the respective networks (FPN, DMN or both) and the ratio between the composite motion score in task state versus resting state) was defined to evaluate whether task-evoked network reconfiguration accounts for a substantial proportion of individual variability in cognitive performance. This was done separately for the two measures of functional connectivity similarity (Pearson correlation between the network graphs and slope of the linear model between task connectivity and rest connectivity). Similarly, linear mixed models with the ratio between the connection strength for the task state and the connection strength for the resting state as predictor were defined to evaluate whether the task-evoked change in connection strength accounts for a substantial proportion of individual variability in cognitive performance.

#### Working memory

In a first set of analyses, accuracy on the N-back task was the dependent variable. For all these models, sex was the only variable that reached statistical significance after backward elimination. The parameter estimates and FDR-adjusted *p*-values of the linear mixed models with task-evoked reconfiguration defined as the Pearson correlation between the network graphs for the different networks as predictor and with the task-evoked reconfiguration defined as the slope of the linear model between task connectivity and rest connectivity for the different networks as predictor are shown in Tables 2 and 3, respectively. The parameter estimates of the linear mixed models with the task-evoked connection strength change for the different networks as predictor are shown in Table 4.

Both measures of similarity of functional connectivity patterns between rest and task pointed in the same direction (although only significant for the slope of the linear model): Lower similarity between resting and task state functional connectivity of the FPN (i.e. more FPN reconfiguration) was associated with a higher working memory score (Fig. 6). No significant association was found for the level of DMN and FPN-DMN reconfiguration. Furthermore, for all networks, changes in connection strength between rest and task were not significantly associated with working memory score (Table 4).

**Table 2:** Parameter estimates of the linear mixed models with the similarity of functional connectivity patterns between rest and task (defined as the Pearson correlation between the network graphs) as predictor for performance on the N-back task

Network	FPN		DMN		FPN-DMN	
Predictor	Beta (standard error)	*p*	Beta (standard error)	*p*	Beta (standard error)	*p*
Similarity rest-task (Pearson correlation)	−17.59 (6.86)	.14	−1.25 (7.59)	.87	−12.03 (7.65)	.28
Sex	3.48 (1.70)	**<.05**	3.85 (1.81)	**< .05**	3.68 (1.76)	**< .05**

**Table 3:** Parameter estimates of the linear mixed models with the similarity of functional connectivity patterns between rest and task (defined as the slope of the linear model between task connectivity and rest connectivity) as predictor for performance on the N-back task

Network	FPN		DMN		FPN-DMN	
Predictor	Beta (standard error)	*p*	Beta (standard error)	*p*	Beta (standard error)	*p*
Similarity rest-task (slope linear model)	−13.16 (4.45)	**< .05**	−3.61 (5.26)	.87	−14.40 (5.59)	.060
Sex	3.87 (1.66)	**< .05**	3.87 (1.80)	**< .05**	3.62 (1.69)	**< .05**

**Table 4:** Parameter estimates of the linear mixed models with the change in connection strength between rest and task as predictor for performance on the N-back task

Network	FPN		DMN		FPN-DMN	
Predictor	Beta (standard error)	*p*	Beta (standard error)	*p*	Beta (standard error)	*p*
Connection strength change	−1.33 (3.49)	.87	1.38 (3.09)	.87	−.68 (3.37)	.87
Sex	3.96 (1.83)	**< .05**	3.85 (1.80)	**< .05**	3.90 (1.83)	**< .05**

BOLD: Significant FDR-adjusted *p*-values

Across all models, being female was associated with a higher working memory score. Note that in none of the models, the composite motion score was included as a significant predictor for the working memory score.

#### Cognitive flexibility

In a second set of analyses, performance on the cognitive flexibility task (raw scores) was the dependent variable. For all these models, the variables frontal tumor involvement and tumor group reached statistical significance after backward elimination. For some models (see Tables 5 and 6), tumor hemisphere was also included after backward elimination. The parameter estimates and FDR-adjusted *p*-values of the linear mixed models with task-evoked reconfiguration defined as the Pearson correlation between the network graphs for the different networks as predictor and with the task-evoked reconfiguration defined as the slope of the linear model between task connectivity and rest connectivity for the different networks as predictor are shown in Tables 5 and 6, respectively. The parameter estimates of the linear mixed models with the task-evoked connection strength change for the different networks as predictor are shown in Table 7.

Both measures of similarity of functional connectivity patterns between rest and task pointed in the same direction (although only significant for the Pearson correlation between the network graphs): lower similarity between resting and task state functional connectivity of the FPN (i.e. more FPN reconfiguration) was associated with a higher cognitive flexibility score (Fig. 7). No significant association with the cognitive flexibility score was found for the level of DMN and FPN-DMN reconfiguration. Furthermore, for all networks, the level of change in connection strength between task and rest was not significantly associated with the cognitive flexibility score (Table 7).

**Table 5:** Parameter estimates of the linear mixed models with the similarity of functional connectivity patterns between rest and task (defined as the Pearson correlation between the network graphs) as predictor for performance on the cognitive flexibility task

Network	FPN		DMN		FPN-DMN	
Predictor	Beta (standard error)	*p*	Beta (standard error)	*p*	Beta (standard error)	*p*
Similarity rest-task (Pearson correlation)	−48.31 (14.29)	**<.05**	−12.24 (16.36)	.52	−41.10 (17.47)	.067
Frontal tumor involvement	−8.41 (4.00)	**<.05**	−11.78 (4.42)	**<.05**	−12.25 (4.12)	**<.05**
Tumor group						
LGG (vs meningioma)	1.30 (4.06)	.89	.70 (4.69)	.89	−1.18 (4.40)	.89
HGG (vs meningioma)	−13.35 (4.57)	**<.01**	−17.59 (5.04)	**<.01**	−16.63 (4.70)	**<.01**
Tumor hemisphere	8.36 (3.47)	.061			9.79 (3.93)	.061

BOLD: Significant FDR-adjusted *p*-values

**Table 6:** Parameter estimates of the linear mixed models with the similarity of functional connectivity patterns between rest and task (defined as the slope of the linear model between task connectivity and rest connectivity) as predictor for performance on the cognitive flexibility task

Network	FPN		DMN		FPN-DMN	
Predictor	Beta (standard error)	*p*	Beta (standard error)	*p*	Beta (standard error)	*p*
Similarity rest-task (slope linear model)	−22.81 (10.14)	.067	−13.64 (11.34)	.38	−32.13 (12.65)	.067
Frontal tumor involvement	−9.81 (4.33)	**<.05**	−11.21 (4.43)	**<.05**	−11.19 (4.07)	**<.05**
Tumor group						
LGG (vs meningioma)	.60 (4.42)	.89	.94 (4.58)	.89	−.65 (4.31)	.89
HGG (vs meningioma)	−15.45 (4.90)	**<.01**	−16.57 (5.05)	**<.01**	−15.46 (4.68)	**<.01**
Tumor hemisphere					9.28 (3.78)	.061

BOLD: Significant FDR-adjusted *p*-values

**Table 7:** Parameter estimates of the linear mixed models with the change in connection strength between rest and task as predictor for performance on the cognitive flexibility task

Network	FPN		DMN		FPN-DMN	
Predictor	Beta (standard error)	*p*	Beta (standard error)	*p*	Beta (standard error)	*p*
Connection strength change	15.26 (7.19)	.071	−2.55 (6.80)	.71	6.44 (7.20)	.48
Frontal tumor involvement	−13.30 (4.27)	**<.05**	−11.78 (4.47)	**<.05**	−12.57 (4.43)	**<.05**
Tumor group						
LGG (vs meningioma)	3.07 (4.50)	.89	1.39 (4.63)	.89	1.93 (4.64)	.89
HGG (vs meningioma)	−17.79 (4.85)	**<.01**	−16.96 (5.22)	**<.01**	−17.89 (5.05)	**<.01**

BOLD: Significant FDR-adjusted *p*-values

Across all models, having a HGG was significantly associated with lower cognitive flexibility. Additionally, a frontal tumor (compared to a non-frontal tumor) was associated with lower cognitive flexibility. The three models in which tumor hemisphere was included pointed in the same direction for the association between tumor hemisphere and cognitive flexibility score: a tumor in the left hemisphere was associated with lower cognitive flexibility (although none of these models reached significance). Note that, again, in none of the models, the composite motion score was included as a significant predictor for the working memory score.

## Discussion

The main goal of this study was to examine whether the level of reconfiguration of the fronto-parietal and default mode network, evoked by task execution, is correlated with cognitive performance in patients with a meningioma or glioma, as has been observed in healthy participants.

In our current study, we found that, across the brain tumor patients, the level of task-evoked reconfiguration of the connections within the FPN was associated with the performance of the working memory task itself as well as with the performance on a cognitive flexibility task, measured outside of the scanner. Furthermore, the level of task-evoked reconfiguration of the connections within and between the FPN and DMN did not differ significantly between meningioma, LGG and HGG patients. Additionally, the changes in strength from rest to task of the connections within and between the FPN and DMN did also not differ significantly between the different tumor groups.

In our study, we observed a relation between the level of reconfiguration of the FPN and executive functioning in brain tumor patients, similar as has been found previously in healthy subjects for working memory (Braun et al. 2015; Vatansever et al. 2017, 2015), for attention (Shine et al. 2016) and for cognitive control (Dwyer et al. 2014). This finding suggests that FPN network reconfiguration not only plays a role in explaining variance in healthy cognitive performance, but also in cognitive deficits in brain tumor patients. A large number of studies have suggested that the FPN is the central control network activated in WM tasks. Other studies have suggested that the FPN also plays an important role in situations requiring highly adaptive task control (Cole et al. 2013; Dosenbach et al. 2006). WM performance is also linked to differences in activation and connectivity within the FPN (e.g. Nagel et al. 2011; Osaka et al. 2004; Ullman et al. 2014). Importantly, our study also indicated that the capacity to reconfigure the connections of the FPN is predictive of cognitive performance on other tasks that engage the same network (Niendam et al. 2012).

Both measures of task-evoked reconfiguration used in this study indicated a positive relation between the level of reconfiguration of the FPN and executive functioning. Using similar reconfiguration measures, other studies on working memory, however, have shown an opposite association between network reconfiguration and cognitive performance (e.g. Schultz and Cole 2016; Vatansever et al. 2015). Based on these findings, Schultz and Cole (2016) suggested that two effects may be at play here, namely an efficiency effect and a distraction-based effect. Their results seem to reflect the efficiency effect which was interpreted as reflecting a more optimal network organization at rest in high performing individuals that supports more efficient (i.e. smaller) topological changes when performing a task. Our results then seem to reflect the distraction-based effect: the higher the network similarity across task and resting state, the poorer the performance. Given that more than half of brain tumor patients indicate to have concentration difficulties (Pranckeviciene et al. 2017), it is indeed reasonable to suggest that our results reflect the distraction-based effect. However, our findings could also be associated with a more general mechanism where network reconfiguration reflects general task-involvement. A low level of reconfiguration would then reflect a low level of task involvement, and consequently poor performance.

Our study also indicated an increase in overall intra-network functional connectivity strength in the FPN and the DMN during task performance in brain tumor patients. In addition, the strength of the inter-network functional connectivity between the FPN and the DMN also increased during task performance. This in line with numerous studies in healthy participants (Ceko et al. 2015; Liang et al. 2016; Newton et al. 2011; Tommasin et al. 2018; Vatansever et al. 2017; Zuo et al. 2018). Note that some studies also showed task-associated reductions of connectivity within the DMN (Gordon et al. 2014; Hampson et al. 2006; Tommasin et al. 2018).

Despite their different origins, infiltration level, malignance and developmental course, task-evoked changes of the connections within and between the FPN and DMN did not differ between meningioma, LGG and HGG patients. This finding is in line with the idea that the impact of all these different brain tumors is not limited to the tumor location itself but spreads to remote brain regions, which fits the network perspective with its focus on connectivity and neural communication across regions. Indeed, focal lesions caused by a glioma (LGG or HGG) have been shown to have widespread effects (e.g. Bosma et al. 2009; Briganti et al. 2012; Park et al. 2016; Xu et al. 2013), even within the hemisphere contralateral to the lesion (e.g. Bartolomei et al. 2006; De Baene et al. 2017; Maesawa et al. 2015). Meningiomas, in contrast with gliomas, do not directly damage the brain regions but yield local effects through perilesional edema and/or mass effect (Whittle et al. 2004). However, these meningiomas might also reduce the functional integrity of remote brain regions (through diaschisis, Carrera and Tononi 2014), as locally compressed brain areas and white matter pathways are densely connected to other parts of the brain.

We also found a significant association between sex and working memory: being female was significantly associated with better performance on the N-back task. This is in line with previous reports showing a female advantage for verbal working memory using the N-back task (Lewin et al. 2001; Speck et al. 2000). Our results also showed a general link between reduced cognitive flexibility and having a HGG. This finding is in line with the observation that rapidly growing, malignant tumors (such as HGG) typically lead to more cognitive impairment than slowly growing tumors (such as meningiomas and LGG) (Hoffermann et al. 2017; Noll et al. 2015; Wilson 1999). Additionally, lower cognitive flexibility was also associated with frontal tumor involvement, which is in line with the fact that cognitive flexibility has been consistently linked with frontal structures (for a review, see Sakai 2008). Similar findings were reported by Hendrix et al. (2017).

A limitation of the current study is that we did not include a healthy control group or longitudinal measures. Therefore, it is impossible to distinguish whether lesion-induced functional changes, compensatory changes, individual differences unrelated to the tumor or a combination of these alter the task-evoked reconfigurations within and between the FPN and the DMN in brain tumor patients. Furthermore, with the inclusion of a healthy control group, we could examine whether the link between the task-evoked reconfiguration of the FPN and cognitive performance is as strong in brain tumor patients as in healthy controls.

Another limitation of the current study is that we examined a group of tumor patients that were very heterogeneous with respect to tumor location. In this study, we only dissociated tumors with a frontal involvement from tumors without a frontal involvement, but we did not take the exact location of the tumor into account. Topological properties of the FPN and DMN might, however, be differently affected depending on the specific region that is lesioned (Yuan et al. 2017). Future studies should, therefore, take the importance of a region in network communication (e.g. hub vs non-hub) into account.

Previous studies have shown that motion during a scan can influence functional connectivity measures (Van Dijk et al. 2012), even after motion estimates have been entered into the regression (Power et al. 2012). Although we used a conservative threshold for data scrubbing, we have examined the effect of motion on our results in several ways. We found no association between behavioral performance and the level of motion during rest or while performing the task. Additionally, in all models, the ratio between the motion score in task state versus resting state was not associated with cognitive performance. This clearly suggests that the findings reported here are not caused by differences in motion.

## Conclusion

In the current study, we found evidence that cognitive performance in brain tumor patients is associated with the capacity to reconfigure the FPN during a cognitive task. This suggests that FPN reconfiguration not only plays a role in the variance in normal cognitive performance in healthy controls, but also in cognitive deficits in brain tumor patients. This finding was independent of the character of the tumor.
